# The Role of P2X7 Purinergic Receptors in the Renal Inflammation Associated with Angiotensin II-Induced Hypertension

**DOI:** 10.3390/ijms21114041

**Published:** 2020-06-05

**Authors:** Rocio Bautista-Pérez, Oscar Pérez-Méndez, Agustina Cano-Martínez, Ursino Pacheco, José Santamaría, Fernando Rodríguez-Sámano, Bernardo Rodríguez-Iturbe, L. Gabriel Navar, Martha Franco

**Affiliations:** 1 Department of Molecular Biology, Instituto Nacional de Cardiología "Ignacio Chávez”, México City 14080, Mexico; rociobtst@yahoo.com (R.B.-P.); oscar.perez.m@tec.mx (O.P.-M.); 2 School of Engineering and Sciences, Tecnológico de Monterrey, Mexico City 14080, Mexico; 3 Department of Physiology, Instituto Nacional de Cardiología “Ignacio Chávez”, México City 14080, Mexico; cmamx2002@yahoo.com.mx; 4 Department of Cardio-Renal Pathophysiology, Instituto Nacional de Cardiología “Ignacio Chávez”, México City 14080, Mexico; upacheco@yahoo.com.mx (U.P.); mulosan@hotmail.com (J.S.); frsyregp@hotmail.com (F.R.-S.); 5 Department of Nephrology, Instituto Nacional de Cardiología "Ignacio Chávez”, México City 14080, Mexico; brodrigueziturbe@gmail.com; 6 Department of Nephrology, Instituto Nacional de Ciencias Médicas y Nutrición “Salvador Zubirán”, Mexico City 14080, Mexico; 7 Department of Physiology and Hypertension and Renal Center, Tulane University School of Medicine New Orleans, New Orleans, LA 70112, USA; navar@tulane.edu

**Keywords:** purinergic receptors, P2X7 receptors, inflammation, kidney, hypertension, angiotensin II

## Abstract

Purinergic receptors play a central role in the renal pathophysiology of angiotensin II-induced hypertension, since elevated ATP chronically activates P2X7 receptors in this model. The changes induced by the P2X antagonist Brilliant blue G (BBG) in glomerular hemodynamics and in tubulointerstitial inflammation resulting from angiotensin II infusion were studied. Rats received angiotensin II (435 ng kg^−1^ min^−1^, 2 weeks) alone or in combination with BBG (50 mg/kg/day intraperitoneally). BBG did not modify hypertension (214.5 ± 1.4 vs. 212.7 ± 0.5 mmHg), but restored to near normal values afferent (7.03 ± 1.00 to 2.97 ± 0.27 dyn.s.cm^−5^) and efferent (2.62 ± 0.03 to 1.29 ± 0.09 dyn.s.cm^−5^) arteriolar resistances, glomerular plasma flow (79.23 ± 3.15 to 134.30 ± 1.11 nL/min), ultrafiltration coefficient (0.020 ± 0.002 to 0.036 ± 0.003 nL/min/mmHg) and single nephron glomerular filtration rate (22.28 ± 2.04 to 34.46 ± 1.54 nL/min). Angiotensin II induced overexpression of P2X7 receptors in renal tubular cells and in infiltrating T and B lymphocytes and macrophages. All inflammatory cells were increased by angiotensin II infusion and reduced by 20% to 50% (*p* < 0.05) by BBG administration. Increased IL-2, IL-6, TNFα, IL-1β, IL-18 and overexpression of NLRP3 inflammasome were induced by angiotensin II and suppressed by BBG. These studies suggest that P2X7 receptor-mediated renal vasoconstriction, tubulointerstitial inflammation and activation of NLRP3 inflammasome are associated with angiotensin II-induced hypertension.

## 1. Introduction

Renal tubulointerstitial inflammation is a consistent feature in angiotensin II-induced hypertension. In short-term angiotensin infusion, the inflammatory infiltration is critical in the subsequent development of salt-sensitive hypertension [1,2,3,4,5], whereas in the chronic angiotensin infusion, tubulointerstitial inflammation is a characteristic that impairs pressure natriuresis, maintains sodium retention and supports blood pressure increment [5]. Tubulointerstitial inflammation precedes glomerular injury [1,6] and its pathogenic relevance has been demonstrated by the reduction of hypertension, with immunosuppressive therapy [7], pentosan polysulphate [8], or genetic manipulations [9].

Purinergic P2 receptors are essential in the regulation of several intrarenal mechanisms that impact the long-term control of blood pressure [4,10,11], such as pressure natriuresis [4,5], autoregulation of glomerular filtration rate and blood flow [12,13], and regulation of sodium excretion. In addition, the activation of purinergic receptors plays an important role in the pathophysiology of angiotensin II (Ang II)-induced hypertension, supporting the production of vasoactive mediators [14,15], sustaining the inflammatory response [16] and impairing pressure natriuresis [5]. The activation of purinergic receptors by elevated concentrations of ATP has been shown in the development of salt-sensitive hypertension and Ang II-induced hypertension [17,18,19]. Higher expression of P2X7 receptors is found in the kidney of Dahl salt-sensitive rats [9] and overexpression of P2X1, P2Y1, P2X4, and P2X7 receptors was described in the intrarenal arteries and afferent arterioles of angiotensin II-infused rats [3,10,19]. In addition, purinergic receptors are central in the ATP-mediated activation of the nucleotide-binding oligomerization domain (NOD), leucine-rich repeat (LRR)-containing protein 3 (NLRP3) inflammasome [20,21,22,23,24,25].

In the model of hypertension induced by Ang II, ATP tubulointerstitial fluid concentrations are elevated [17], associated with activation of P2 receptors [17]. Moreover, previous studies have shown that the administration of P2X7 antagonists prevents the development of renal inflammation [9].

The purpose of this study was to characterize P2X7 receptors in the inflammatory cells infiltrating tubulointerstitium in Ang II-induced hypertension, to evaluate proinflammatory cytokine release and activation of the NLRP3 inflammasome, as well as to study the effects resulting from the chronic administration of a P2X7 antagonist in the renal function, glomerular hemodynamics and tubulointerstitial inflammation.

## 2. Results

### 2.1. Systolic Blood Pressure and Proteinuria

**Figure 1:**
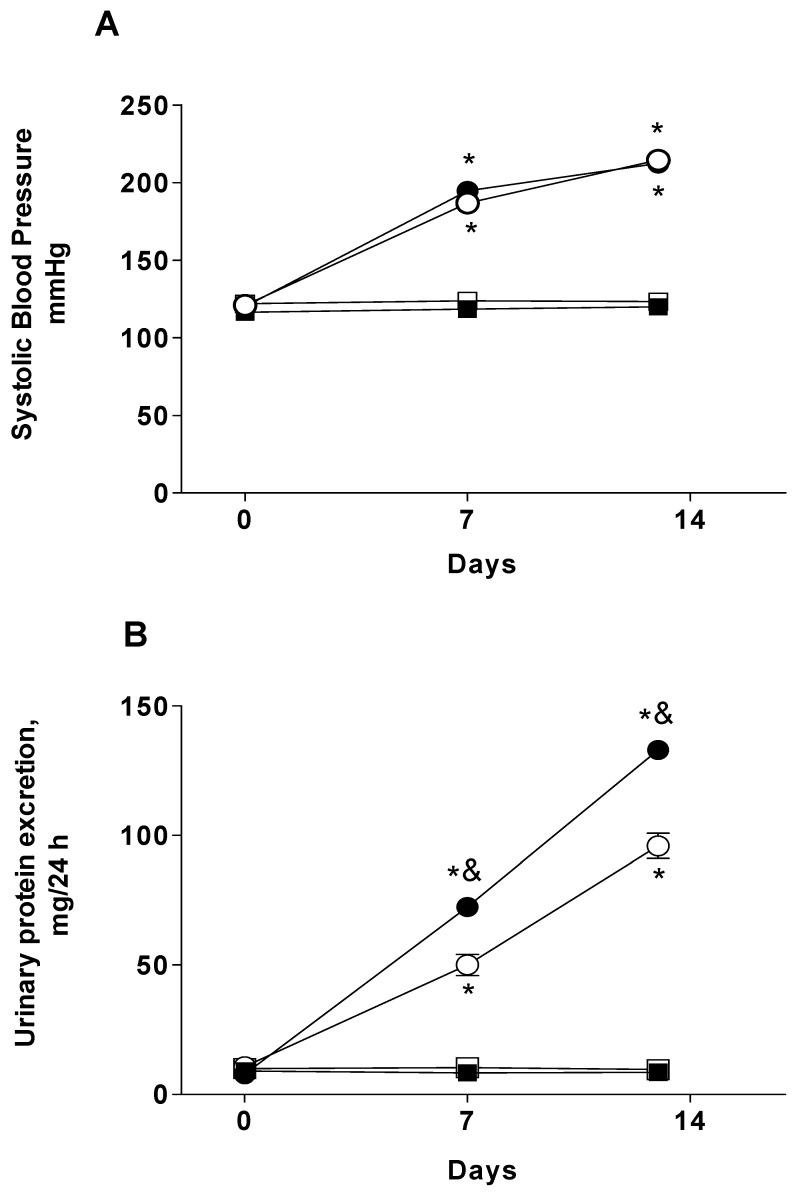
Systolic blood pressure (**A**) and urinary protein excretion (**B**) in Sham-operated, Sham + BBG, Ang II, and Ang II + BBG groups. Black squares ■ Sham; open squares □ Sham + BBG; black circles ● Ang II; open circles ◯ Ang II + BBG; *n* = 8 rats/group. * *p* < 0.001 vs. Sham; & *p* < 0.05 vs. Ang II + BBG. Ang II = angiotensin II, BBG = Brilliant blue G.

After the implant of the miniosmotic pump, angiotensin II induced a progressive increase in systolic blood pressure in rats (Figure 1A). The blood pressure increment was similar in the Angiotensin II group (Ang II) and in the Ang II + BBG group, reaching around 200 mmHg at the end of the experiment (day 14), while sham rats remained normotensive (Figure 1A). Urinary protein excretion gradually increased in the rats that received the angiotensin II infusion. This effect was significantly higher in the Ang II group (140.60 ± 2.61 mg/day at day 14) than in the Ang II+BBG group (80.00 ± 4.34 mg/day) (Figure 1B).

### 2.2. Micropuncture Studies

**Figure 2:**
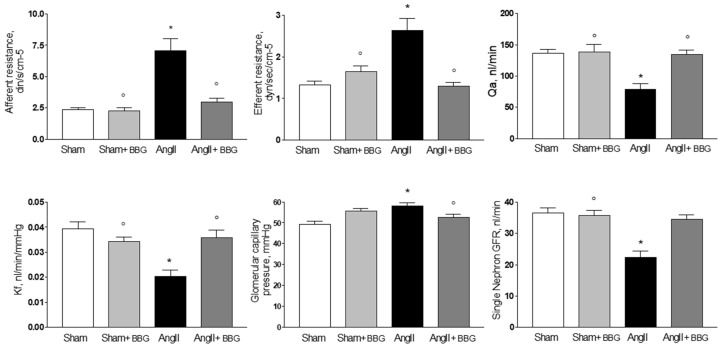
Glomerular hemodynamics in Sham, Sham + BBG, Ang II and Ang II + BBG groups (*n* = 8 rats/group). * *p* < 0.001 vs. Sham; ^◯^ *p* < 0.001 vs. Ang II. Qa = glomerular plasma flow; Kf = ultrafiltration coefficient; GFR = glomerular filtration rate, Ang II = angiotensin II, BBG = brilliant blue G.

Concerning glomerular hemodynamics, Ang II-infused rats exhibited increased afferent (AR) and efferent (ER) arteriolar resistances, which resulted in reduction of glomerular plasma flow (Qa), as previously reported [3]. The ultrafiltration coefficient (Kf) and single-nephron glomerular filtration rate (SNGFR; Figure 2) were also reduced by Ang II. The whole kidney glomerular filtration rate (GFR), was lower. Administration of BBG for 14 days reduced the arteriolar vasoconstriction induced by chronic angiotensin II infusion (Figure 2).

Afferent and efferent resistances were both significantly reduced with BBG administration (58% and 51%, respectively). The reduction on resistances led to a greater glomerular plasma flow (Qa). 134.30 ± 1.1 nL/min in Ang II + BBG vs. 79.23 ± 3.15 nL/min in Ang II rats (*p* < 0.05). The ultrafiltration coefficient (Kf) was lower in the Ang II group than in the Ang II+BBG (0.020 ± 0.002 nL/min/mmHg, and 0.036 ± 0.0030 nl min mmHg, respectively (*p* < 0.05). A similar pattern was observed in the single-nephron glomerular filtration rate (SNGFR), which was greater in the group treated with BBG (34.46 ± 1.54 nL/min) than in the in Ang II without BBG (22.28 ± 2.04 nL/min, *p* < 0.05) (Figure 2). Mean arterial and glomerular capillary pressures (PGG; Figure 2) were not altered by the treatment. In sham-operated rats, BBG did not elicit significant changes either in glomerular hemodynamics (Figure 2), or in whole kidney glomerular filtration rate (GFR); Sham and Sham + BBG had similar GFR (1.19 ± 0.056 vs. 1.09 ± 0.067 mL/min, respectively), suggesting a minor activity of P2X7 receptors in physiological normotensive states. In the Ang II + BBG group, GFR was higher (0.98 ± 0.062 mL/min) than in the Ang II + vehicle (V) group (0.72 ± 0.055 mL/min, *p* < 0.05),

### 2.3. Histological Analysis

**Figure 3:**
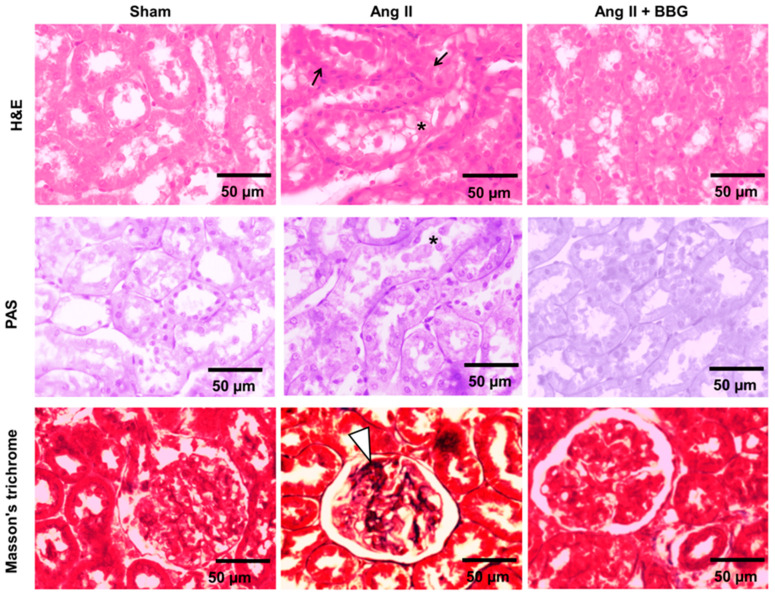
Representative histological microphotographs stained with hematoxylin and eosin (**H&E, first row**), periodic acid Schiff (**PAS, second row**), and Masson´s trichrome (**third row**) in renal cortex of rats in Sham, Ang II and Ang II + BBG groups (*n* = 7 per group). In the Ang II group, there are areas of tubulointerstitial cell injury with intratubular debris indicated with an asterisk (*), focal areas of mononuclear infiltration indicated by black arrows and modest segmental mesangial widening in the glomeruli (white arrow head, ∇) are reduced in the Ang II + BBG group. Ang II = angiotensin II; BBG = brilliant blue G.

The analyses with hematoxylin and eosin (H&E), periodic acid Schiff (PAS) and Masson´s trichrome staining were performed to evaluate the histological renal changes induced by Ang II, as well as the modifications associated with the administration of BBG. Renal tissue obtained at the end of the Ang II infusion (day 14) showed tubulointerstitial cell injury with intratubular debris and focal areas of mononuclear infiltration (Figure 3), as well as modest segmental mesangial widening in the glomeruli; these findings agreed with previous reports [1]. Co-administration of BBG treatment with Ang II was associated with histological improvement (Figure 3).

### 2.4. Immune Cell Infiltration and P2X7 Protein Expression

**Figure 4:**
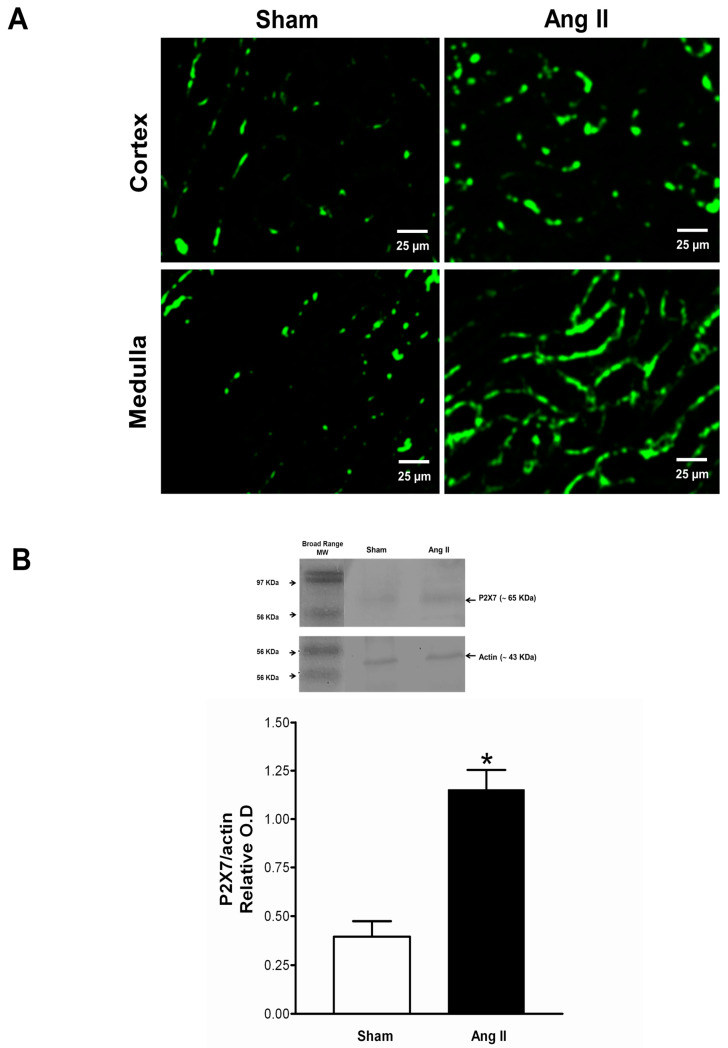
P2X7 receptors identified by immunofluorescence, in renal cortex and medulla in Sham and Ang II-infused rats. The expression of P2X7 receptors in the Ang II group is localized in tubular membranes (**A**). Western blot (**B**) showed abundance of the P2X7 receptor protein in the Ang II group (*n* = 7 per group). * *p* < 0.01 vs. Sham. Ang II = angiotensin II, BBG = Brilliant blue G.

These studies were performed in additional groups of seven Sham-operated and seven Ang II-infused rats. One kidney was used for immunofluorescence and the other for Western blot analysis. Figure 4 (upper panel) shows an overexpression of P2X7 receptors in tubular cells in the cortex and medulla of Ang II-infused rats, contrasting with the negative staining of the sham-operated rats. P2X7 receptors are localized in tubular membranes as previously described [24,26]. Concordant results were found with Western blot (Figure 4, bottom panel).

**Figure 5:**
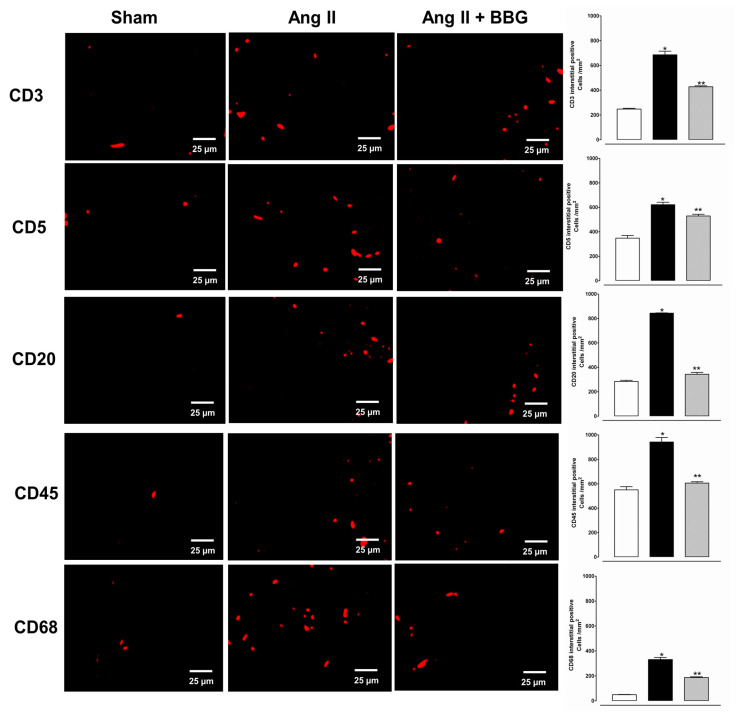
Infiltrating T cells (CD3, CD5), B cells (CD 20) and macrophages [CD68], are increased in the renal cortical interstitium of the angiotensin-infused rats (Ang II group *n* = 7), and reduced by the administration of BBG (Ang II + BBG *n* = 7). CD45 antigen is a leukocyte common antigen predominantly expressed in lymphocytes. White bars (□) Sham; Black bars (■) AngII; and grey bars (■) Ang II + BBG. * *p* < 0.001 vs. sham, ** *p* < 0.05 vs. Ang II. Ang II = angiotensin II; BBG = brilliant blue G.

Two weeks of angiotensin infusion induced an intense infiltration of T lymphocytes, B lymphocytes and macrophages in the kidney (Figure 5). The inflammatory infiltration was diminished by BBG administration.

**Figure 6:**
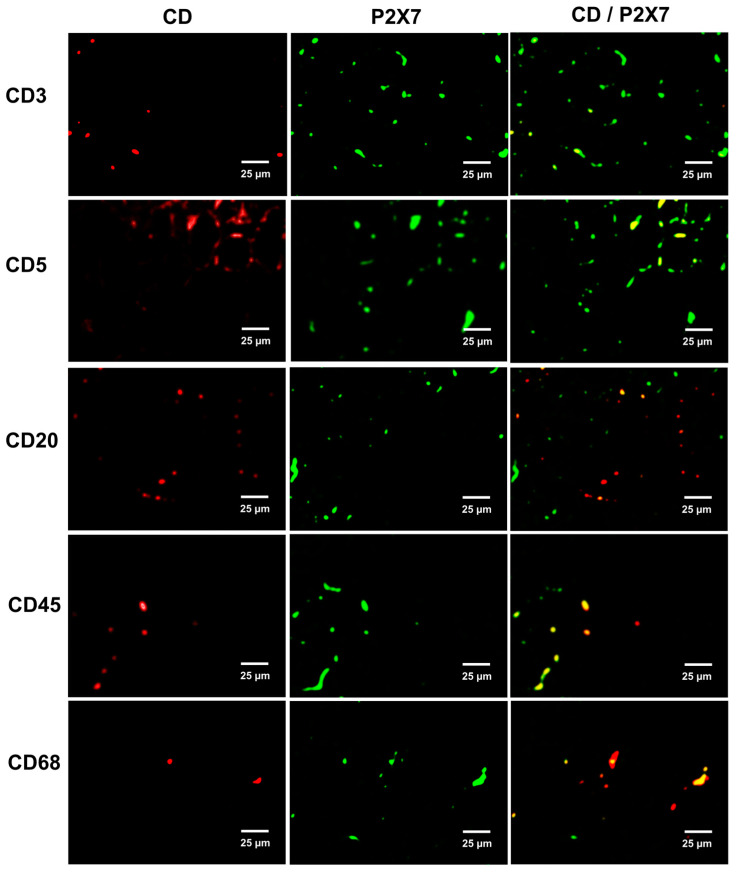
P2X7 receptors (second column) in the infiltrating T cells of Ang II group co-localize with cluster differentiation (CD, first column) antigens in T cells (CD 3 positive cells), B cells (CD5 and CD20 positive cells), macrophages (CD68 positive cells) and leukocyte common antigen CD45, predominantly expressed in T lymphocytes.

P2X7 receptors are present in the infiltrating cells in the kidney of the angiotensin II-infused rats. Figure 6 shows the co-localization of P2X7R and CD3, CD5, CD20, CD68 and CD45 antigens identifying T cells (CD3 and CD5 positive cells), B cells (CD20 positive cells), macrophages (CD68 positive cells) and CD45 (leukocyte common antigen predominantly expressed in lymphocytes).

### 2.5. Interleukins, TNFα and NLRP3 Inflammasome

**Figure 7:**
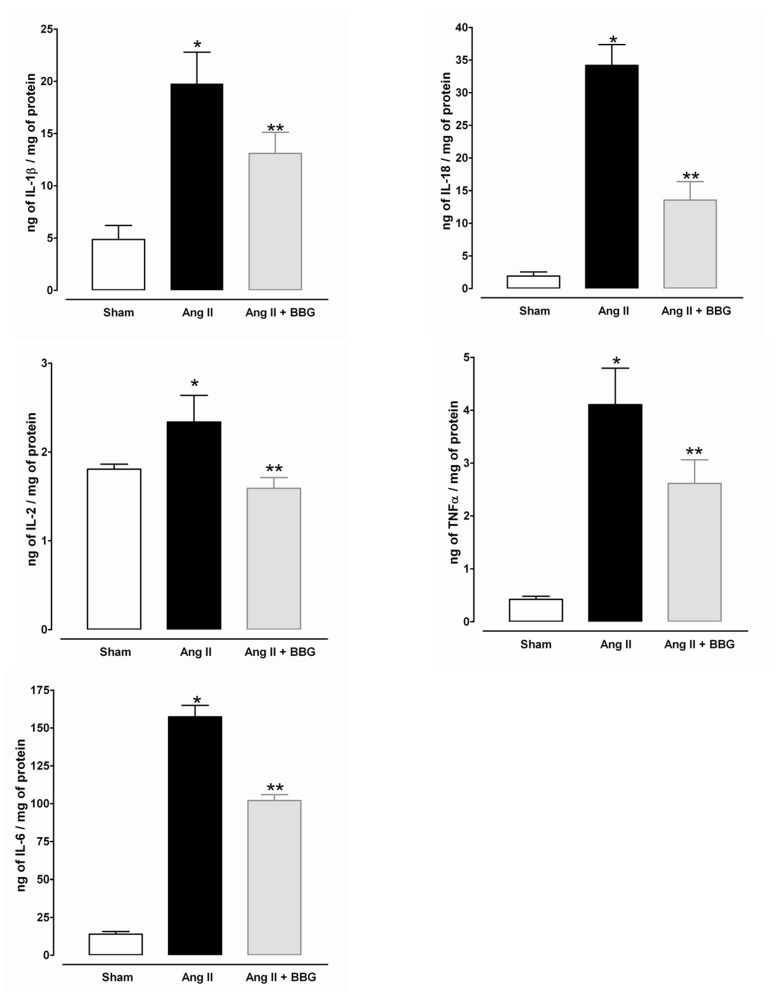
Interleukins IL-1β, IL-2, IL-6, IL-18 and TNFα in the renal cortical tissue from Sham, Ang II and Ang II + BBG, (*n* = 7 per group). * *p* < 0.001 vs. Sham, ** *p* < 0.05 vs. Ang II. Ang II = angiotensin II; BBG = brilliant blue G.

Interleukins IL-1β, IL-2, IL-6, IL-18, and TNFα, were all increased as a result of Ang II infusion (*p* < 0.001), whereas P2X7 blockade resulted in a significant decrease in the expression of each of these proteins without reaching the levels in the sham-operated group (Figure 7).

**Figure 8:**
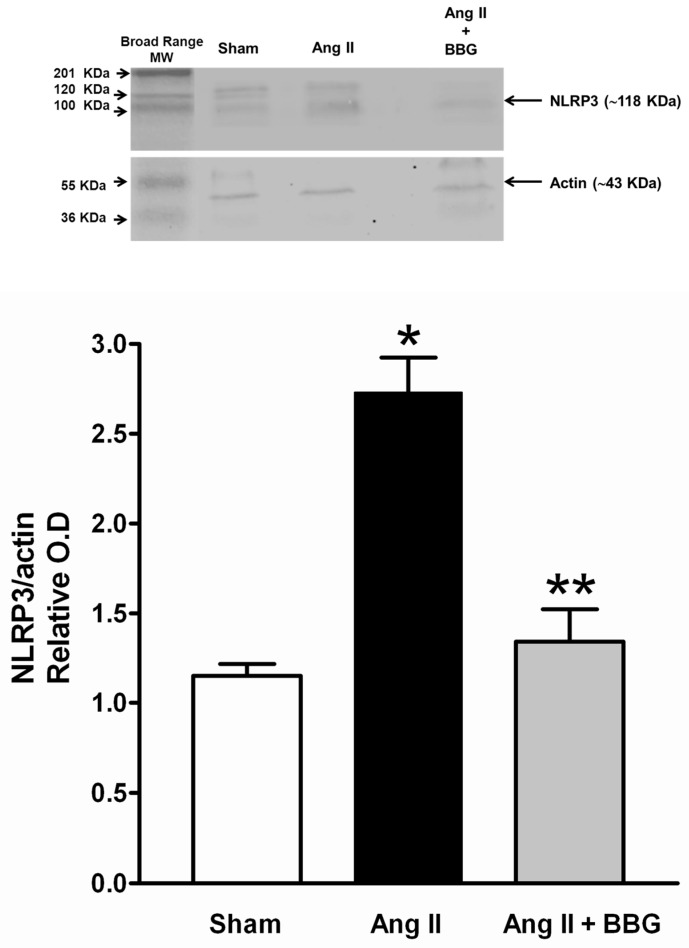
NLRP3 determined by WB in Sham, Ang II and Ang II + BBG. NLRP3 was increased with Ang II and significantly decreased with the administration of the antagonist. * *p* <0.05 vs. Sham; ** *p* < 0.05 vs. Ang II. NLRP3 = (nucleotide-binding oligomerization domain (NOD), leucine-rich repeat (LRR)-containing protein 3) inflammasome; Ang II = angiotensin II; BBG = brilliant blue G.

Angiotensin II-induced overexpression of NLRP3 (nucleotide-binding oligomerization domain (NOD), leucine-rich repeat (LRR)-containing protein 3) inflammasome protein that was suppressed by the blockade of P2X7 with BBG (Figure 8).

## 3. Discussion

It has been well established that hypertension, proteinuria and tubulointerstitial inflammation are the result of Ang II infusion [1,25]. These abnormalities are likely mediated by the coexistent activation of angiotensin II receptors and purinergic receptors, since both receptors are elevated in the kidney [1,21]. In this study, severe hypertension, with significant proteinuria and renal injury, developed after two weeks of Ang II infusion, as expected [19].

In contrast to other reports (9, 18), the administration of BBG did not modify the hypertensive response to Ang II, but significantly reduced interstitial inflammation and proteinuria [9,18]. Differences in the studies may explain the lack of blood pressure reduction in the present investigation. Studies that found amelioration of hypertension with P2X7 purinergic receptor blockade were done in F344 rats that overexpress P2X7 receptors in the kidney, and used smaller doses of angiotensin II (30ng/kg/min) that resulted in a lesser increment in blood pressure [10]. We selected a higher dose of Ang II (435 ng/kg/min) because our interest was focused in the inflammatory tubulointerstitial infiltration [19,22]. BBG is a non-selective purinergic receptor antagonist with a high and predominant antagonistic potency against the P2X7 receptor. BBG produces a non-competitive inhibition of rat P2X7 receptors with IC_50_ values of 10 nM. IC_50_ values of BBG for inhibition of P2X2 receptors was 1.5 and 10 μM for P2X4 receptors [23]. Whether P2X4 receptors were blocked in the previous studies by our group, the specific blockade of P2X4 receptors had minor effects in renal hemodynamics induced by angiotensin II [19], while the blockade obtained with the specific P2X7 antagonist A438079 was strong and similar to the response induced by BBG (19). Supported by this evidence, we were confident that BBG effects on glomerular hemodynamics were the result of P2X7R antagonisms [18,19]. P2X2 receptors and Na^+^ channels could also be blocked by BBG; however, no vasoactive effects have been reported for the blockade of these receptors or on sodium channels.

In the present study, chronic P2X7 blockade with BBG restored vascular resistances glomerular blood flow, ultrafiltration coefficient, and single nephron glomerular filtration rate to near-control values. A similar effect was observed in total GFR. The restoration of glomerular hemodynamics to near-normal values by BBG is in accordance with previous studies showing overexpression of P2X7 receptor in the renal vessels in the Ang II-induced hypertension [19]. A small but significant increase in glomerular capillary pressure caused by P2X7R blockade can be the result of a greater reduction of the afferent than of the efferent resistances. Previous studies have also found predominant afferent arteriole effects of purinergic agonists [25]. The decrease in efferent resistance in Ang II-induced hypertension when treated with BBG suggests the presence of purinergic receptors in efferent arterioles. An alternative explanation to the observed reduction in efferent resistance is the potential activation of adenosine A2 receptors [26]. ATP is metabolized to adenosine, which is elevated in Ang II-induced hypertension [14]. Activation of adenosine A2 receptors elicits efferent arteriolar vasodilation in in vitro-constricted efferent arterioles [27]. Although consistent with previous data using non-selective P2 blockers, the dominance of the P2 receptors to regulate glomerular arteriole tone, suggests a complex interrelation between AT1 receptors and purinergic receptors in conditions of increased ATP and Ang II levels [18,19,27]. The AT1R blockade reduces glomerular afferent and efferent arteriolar resistances [28], but the extent to which AT1R blockade reduces the glomerular arteriolar resistances in sustained hypertension has not been completely studied. It is possible that AT1R and P2XR share common post-receptor signaling, which would allow a substantial overlap in their actions to control glomerular hemodynamics in hypertension [29].

Ang II infusion resulted in overexpression of P2X7R in the kidney and in the infiltrating cells. The P2X7R is considered a proinflammatory receptor and its location in renal tubular tissues and inflammatory cells suggests that both tubular and immune cells are involved in the proinflammatory reactivity that generates immune cell accumulation [18,19,30].

Inflammatory cells have P2X1, P2X4, P2X7 and P2Y membrane receptors. ATP stimulates purinergic receptors in resident inflammatory and renal tubular cells that attract circulating inflammatory cells [30]. In addition, ATP activates the receptor and induces the assembly of the NRLP3 inflammasome [31], resulting in the generation and release of IL-18 and IL-1β and the development of local inflammatory reactivity [32,33]. We reasoned that the high potency of the antagonism of BBG for P2X7 receptors would permit the evaluation of the predominant role of these purinergic receptors in the inflammation induced by angiotensin II [34,35]. In fact, BBG treatment reduced immune cell infiltration, inflammatory cytokine generation, and NLRP3 inflammasome expression.

Since BBG is a non-selective antagonist of P2X7 receptors, the present study cannot exclude the participation of the blockade of other P2 receptors in the observed results; however, as discussed earlier, it is reasonable to assume that most, if not all, the observed effects of BBG administration are the result of P2X7 antagonism. Another limitation of the study is that signaling pathways of P2X7 receptors were not investigated.

The reduced expression of P2X7 receptors induced by BBG is likely the result of reduced inflammation. The overexpression of P2X7 receptors resulting from angiotensin II infusions is mediated by increased ATP stimulation. Reduced inflammation would decrease ATP activation and thereby diminish the expression of P2X7 receptors [36].

The histological analysis is in concordance with previous studies that showed tubulointerstitial inflammation is a result of Ang II infusion [22,37], and present studies showed that reduction of the inflammatory infiltration is a result from BBG administration. Previous studies have identified the role of tubulointerstitial inflammation in the pathogenesis of salt-sensitive hypertension [38] and showed that a variety of immunosuppressive treatments prevent and correct hypertension [6,7,8]. It is likely that the reduction of the inflammatory infiltrate induced by P2X7R blockade would have a similar effect on salt-driven hypertension [33]. The blockade of P2X7R in lymphocytes, macrophages and dendritic cells may have so far unexplored effects on the immune response that play a recognized role in the pathogenesis of renal injury and hypertension [30]. While direct evidence of Ang II-driven NLRP3 activation was not obtained, the increase in IL1β and IL-18 and NLRP3 expression induced by angiotensin II infusion and their suppression by P2X7R blockade is consistent with this possibility [32,35,39]. Interestingly, BBG treatment reduced the protein expression of NLRP3 induced by Ang II administration which is also consistent with the possible role of P2X7 receptors in inflammasome activation.

In summary, this study indicates that hemodynamic alterations and renal inflammation resulting from angiotensin II infusion are associated with an overexpression of P2X7 receptors. BBG administration improves glomerular hemodynamics, reduces immune cell infiltration, proinflammatory cytokines and NLP3 expression, as a result, at least in part, of the P2X7 receptor blockade.

## 4. Materials and Methods

### 4.1. Animal Procedures

Animal procedures were performed in accordance with the Mexican Federal Regulation for animal experimentation and care (NOM-062-ZOO-2001) and protocols were approved by the Investigation Committee of the Instituto Nacional de Cardiología “Ignacio Chávez”, (INC-CICUAL/012/2019, 17-1142). All rats had free access to water and standard chow diet.

Male Wistar rats (350–360 g) received a two-week infusion of ANG II (Sigma, St. Louis, MO, USA) via subcutaneous osmotic minipumps (Alzet 2002; Alza, Palo Alto, CA) implanted under isofluorane anesthesia. Minipumps delivered Ang II at a rate of 435 ng kg^−1^ min^−1^ [20]. In the corresponding experimental groups, rats received either vehicle or the non-selective P2X7 blocker brilliant blue G (BBG) (50 mg/kg/day), given intraperitoneally during the Ang II infusion. The dose of BBG was selected on the basis of previous studies that have demonstrated that this dose corrects P2X7R-mediated interstitial inflammation and salt-driven hypertension [18].

#### 4.1.1. Design of the Experiments

Experimental groups consisted of sham-operated (*n* = 8) and Ang II-infused rats that received vehicle (Ang II group *n* = 8) or BBG (Ang II + BBG group, *n* = 8); Rats were euthanized 13-14 days after the implantation of osmotic minipumps for Ang II infusion. Micropuncture studies were done in additional sham-operated rats (*n* = 8) and rats of the Ang II group (*n* = 8) as well as Ang II +BBG group (*n* = 8).

#### 4.1.2. Micropuncture Studies

Animals were anesthetized with sodium pentobarbital (30 mg/kg, i.p.) and additional intravenous doses were administered as required. The rats were placed on a thermo-regulated table and the temperature was maintained at 37 °C. Polyethylene tubing (PE-240) was used to catheterize the trachea to maintained breathing, jugular veins to fluid infusion, right femoral artery (PE-50) to continuously measure mean arterial pressure (MAP) and the left ureter (PE-10). The left kidney was exposed and placed in a lucite holder with Ringer solution covering the kidney surface. During the surgery, rats received a plasma infusion (1% of body weight) through a jugular catheter. Immediately afterward, a bolus injection of 100 mg of polyfructosan in 0.5 mL of Ringer’s solution, and an infusion of 10% polyfructosan in Ringer’s solution was started at a rate of 2.2 mL/h (Inutest; Fressenius Pharma, Austria). After 60 min, seven timed samples of proximal tubular fluid were obtained to determine tubular flow and polyfructosan concentration. Intratubular hydrostatic pressures under free-flow and stop-flow conditions were measured in other proximal tubules, and peritubular capillary pressures were also measured using a Servonull device (Servo-Nulling Pressure System; Instrumentation for Physiology and Medicine, San Diego, CA, USA) as previously described [40,41]. MAP was continuously monitored with a pressure transducer (Gould-Statham Instruments, Hato Rey, PR) and recorded on a polygraph (Grass Instruments, Quincy, MA, USA). Blood samples were taken periodically and replaced with an equal volume of resuspended red blood cells in saline solution. Polyfructosan concentrations were determined in plasma and urine samples using a method described by Davidson et al. [42]. Glomerular colloid osmotic pressures were estimated using the plasma protein concentrations in blood taken from the femoral artery and from surface efferent arterioles as previously described [40]. The volume of fluid collected from an individual proximal tubule was estimated from the length of a capillary tube of uniform bore and known internal diameter. The concentration of tubular polyfructosan was measured using the method described by Vurek and Pegram [43]. The plasma protein concentrations of samples obtained from the efferent arterioles and from femoral artery were determined using the method described by Viets et al. [44]. Proximal single nephron glomerular filtration rate, intratubular pressure during free-flow conditions and under stopped-flow conditions (after blocking the tubular lumen with a long oil column), glomerular capillary hydrostatic pressure, peritubular capillary pressure, afferent oncotic pressure, efferent oncotic pressure, glomerular capillary hydrostatic pressure gradient, single-nephron filtration fraction, single-nephron Qa, afferent and efferent resistances (AR and ER, respectively), Kf, and oncotic pressure were measured or calculated according to equations previously described [41,45].

Arterial pressure measurements in conscious rats: Systolic blood pressure (SBP) measurements were performed in conscious, restrained rats by tail-cuff plethysmography (NIBP System, PanLab, Barcelona, Spain). The rats were conditioned three times before blood pressure were measured at a basal period and every week. For proteinuria assessment, rats were placed in metabolic cages with water and food available ad libitum, and urine was collected for a 24-h period; the collections were taken during a basal period and every week before the experiments. Urine samples were used for determination of proteinuria. Urinary protein excretion was measured by the trichloroacetic acid assay [46] using bovine serum albumin as the protein standard.

#### 4.1.3. Immunofluorescence Studies

These studies were performed in additional groups of seven sham-operated, seven Ang II-infusion, and seven Ang II + BBG rats. One kidney was used for immunofluorescence, and the other for Western blot analysis. Kidney sections were incubated with 10% blocking serum in PBS for 1 h at room temperature followed by incubation at 4 °C overnight with the fluorescein isothiocyanate (FITC)-labeled antibodies anti-P2X7-FITC (Cat #: APR-008-F, rabbit polyclonal antibody, Alomone Laboratories, Jerusalem, Israel) at 1:250 [47]. Tissues were co-stained with phycoerythrin (PE)-labeled antibodies, anti-CD3-PE, -CD5-PE, -CD20-PE, -CD45-PE, or -CD68-PE antibodies (Cat #: sc-20047 PE, sc-1180 PE, sc-393894 PE, sc-1178 PE, sc-20060 PE, mouse monoclonal antibodies Santa Cruz Biotechnology, Dallas TX, USA), for localization of receptors expressed in inflammatory cells. To reduce autofluorescence from other sources, such as collagen, elastin, red blood cells, and general background fluorescence, the samples were treated with TrueBlack Lipofuscin Autofluorescence Quencher (#23007; Biotium, TermoFisher, Waltham, MA, USA) [48] Negative controls were performed using an identical protocol but excluding the primary antibody.

Tissues were mounted on microscope coverslips using Vectashield. Signals were examined under a Cell Imaging Station (Life Technologies, Carlsbad, CA). Analysis was performed on 10 fields/section (magnification ×200). Results are presented as mean number of positive cells per field.

#### 4.1.4. Western Blot for P2X7 Receptor and NLRP3 Proteins

Whole kidney tissue was frozen in liquid nitrogen and stored at -70 °C until used. Proteins (40 µg) were separated in 7.5% SDS-PAGE and transferred onto a nitrocellulose membrane. Membranes were incubated overnight with primary antibody P2 × 7 1:500 (rabbit polyclonal, Cat #: APR-008, Alomone Laboratories, Jerusalem, Israel) or NLRP3 1:500 (rabbit polyclonal, Cat # ab214185, Abcam, Cambridge, MA, USA), followed by HRP-labeled secondary antibodies (goat anti-rabbit IgG- HRP, Cat # ab205718 Abcam, Cambridge, MA, USA) for 2 h. To estimate the molecular weight (MW) of sample proteins, we used Strep Tactin horse radish peroxidase conjugate (Cat #: 1,610,380 Biorad, California, USA). The blots were visualized with an enhanced chemiluminescent system (Amersham Biosciences, Buckinghamshire, UK). The acquisition image was performed with a ChemiDoc Imaging System (Bio-Rad. Philadelphia PA USA,) and densitometric quantification of antibody-specific bands was performed with the Image Lab software. Each membrane was stripped of bound antibody and re-probed with anti-actin HRP-labeled antibody (1:1000, Santa Cruz Biotechnology Dallas TX, USA) on the same membrane for quantitative comparison [49].

#### 4.1.5. Cytokines Determination

The concentration of cytokines IL-1β, IL-2, IL-6, IL-18, and TNF-α were determined in kidney cortex protein extracts. Cytokines were quantified by a magnetic bead panel (Millipore, Billerica, MA) according to kit instructions and analyzed on a MagPix instrument (Luminex Corporation, Austin TX). Each sample was measured in duplicate. To perform cytokine analysis, the total protein concentration was quantified using the Bradford assay and diluted to a final protein concentration of 1 μg/μL.

#### 4.1.6. Histologic Analyses

The analyses with hematoxylin & eosin (H&E), periodic acid Schiff (PAS) and Masson’s trichrome staining were performed for the evaluation by light microscopy of the renal histological changes induced by Ang II, as well as the modifications observed with the administration of BBG. Tissues were sectioned into 4–6 μm slices and stained with hematoxylin and eosin (H&E), periodic acid-Schiff (PAS), and Masson’s trichrome. The morphological structure of cortical tissues samples was observed under a light microscope, and photomicrographs were taken with an Olympus BX51 and Q-Imaging publisher 5.0 RTV.

#### 4.1.7. Statistical Analysis

Statistical analysis was performed using Prism software (Graph-Pad, San Diego CA). Results are presented as means ± SE. The significance of differences between groups was evaluated by ANOVA followed by the Tukey post hoc test for multiple groups. Differences with *P* < 0.05 were considered statistically significant.

## Abbreviations

Ang II	Angiotensin II
AR	Afferent resistance
ATP	Adenosine triphosphate
BBG	Brilliant blue G
ER	Efferent resistance
GFR	Glomerular filtration rate
H&E	Hematoxylin and eosin staining
Kf	Ultrafiltration coefficient
Qa	Glomerular plasma flow
PAS	Periodic acid Schiff staining
P2 × 1	Purinergic P2 × 1 receptors
P2 × 4	Purinergic P2 × 4 receptors
P2 × 7R	Purinergic P2 × 7 receptors.
P2Y1	Purinergic P2Y1 receptors
SNGFR	Single-nephron glomerular filtration rate
